# Estrogen receptors mediate the antidepressant effects of aerobic exercise: A possible new mechanism

**DOI:** 10.3389/fnagi.2022.1040828

**Published:** 2022-12-09

**Authors:** Ruixue Zhou, Zhisheng Wang, Bojun Zhou, Zixin Yu, Chongyun Wu, Jun Hou, Ken Cheng, Timon Chengyi Liu

**Affiliations:** School of Physical Education and Sports Science, South China Normal University, Guangzhou, Guangdong, China

**Keywords:** aerobic exercise, anxiety-depression-like behavior, chronic restraint stress, estrogen depletion, estrogen receptor antagonists, estrogen replacement therapy

## Abstract

**Purpose:**

This study aimed to examine whether aerobic exercise exerts mood-modulating effects through an estrogen signaling mechanism.

**Method:**

The experiment was divided into two parts. The first part is to compare the three modeling methods to obtain the most obvious method of depression-like phenotype for further study in the second part. The first part of ovariectomized rats (age, 13 weeks) was tested when rats were 14 or 22 weeks old or in the sixth week after 3 weeks of chronic restraint stress. The second part was to treat the animals with the most obvious depression-like phenotype in different ways, placebo treatment or estradiol (E2) replacement therapy was administered, aerobic training, or estrogen receptor antagonist treatment. The cognitive (Barnes maze and 3-chamber social tests), anxiety-like (open-field and elevated plus maze tests) and depression-like (sucrose preference and forced swim tests) behaviors of rats in both parts were analyzed to study the effects of estrogen depletion and aerobic exercise.

**Results:**

Rats did not develop depressive symptoms immediately after ovariectomy, however, the symptoms became more pronounced with a gradual decrease in ovarian hormone levels. Compared with the placebo or control groups, the exercise and E2 groups showed improved performance in all behavioral test tasks, and the antidepressant effects of aerobic exercise were comparable to those of estrogen. Moreover, the estrogen receptor antagonist has markedly inhibited the antidepressant effects of aerobic exercise.

**Conclusion:**

Estrogen receptors may mediate the antidepressant effects of aerobic exercise. In addition, an increasingly fragile ovarian hormonal environment may underlies chronic restraint stress-induced depression.

## Introduction

Major depressive disorder (MDD) is a common neuropsychiatric disorder that consistently ranks high in terms of disability-adjusted life years worldwide (GBD, 2016). It is characterized by persistent spontaneous depressed mood, loss of interest, appetite/weight disturbance, sleep disturbance, altered mental status, decreased energy levels, feeling of worthlessness, lack of concentration/indecision, and suicidal ideation (Zimmerman et al., 2015). MDD seriously threatens the life of patients, deprives patients of basic social functions, and imposes a huge economic burden on the family and society.

Depression is very common throughout the human life span. From adolescence onward, women are 1.5–3 times more likely to develop various depressive disorders than men (García-Portilla, 2009). Studies have reported that the incidence of depression in women increases during phases of hormonal transitions, such as adolescence, pregnancy and menopause (Steiner et al., 2003). Hormones, also known as sex steroids, such as estrogen and progesterone, are primary substrates for the development of depression in women. In addition to the direct influence of sex hormones, the interaction between sex steroids and the hypothalamic–pituitary–adrenal (HPA) axis, which is a major stress response system in the body, may promote susceptibility to depression. Stress (Bourke et al., 2012; Gobinath et al., 2014), diseases (Nemeth et al., 2013) and other experiences can increase sex-specific susceptibility to depressive episodes. This phenomenon may be partly attributed to the role of steroid receptors, which act as nuclear receptors, as transcription factors that can manipulate gene expression by exerting profound and long-lasting effects on physiological conditions and behavior (Bourke et al., 2012).

In women, ovarian hormones play an important role in developing susceptibility to depressive states (Silberg et al., 1999; Sanchez et al., 2010; Bourke et al., 2013; Melas et al., 2013) and potentially contribute to changes in the therapeutic effects of drugs and other modalities through developmental changes at the tissue level (Brunton and Russell, 2010; Doyle et al., 2015; Sickmann et al., 2015; Braithwaite et al., 2017), acute activation changes (Kessler, 2003; Van den Bergh et al., 2008)and interactions with other steroid systems (Martinez et al., 2016; Bixo et al., 2018). Reproductive ageing is characterized by major hormonal shifts. During perimenopause, women experience a regression in the levels of ovarian hormones, a change that may be accompanied by a depressed mood (Kessler, 2003). In addition, depressive episodes during the menopausal transition can be more pronounced than those manifested in early life (Bromberger and Kravitz, 2011; Maki et al., 2019; Tang et al., 2019). Therefore, fluctuations in the levels of ovarian hormones may increase the risk of depression during the menopausal transition among women, which is consistent with the ovarian steroid withdrawal hypothesis (Shaikh, 1971; Bloch et al., 2000; Galea et al., 2001). Furthermore, the history of MDD is associated with an earlier decline in ovarian function (Finch, 2014), suggesting that the relationship between ovarian hormones and depression may be bidirectional.

Studies have demonstrated that depression during the menopausal transition is responsive to hormones. Treatment with 17β-estradiol for 4 weeks can alleviate anxiety-like behaviors (Renczés et al., 2020), attenuate stress-induced exploratory and anxiety-like behavioral impairment and improve spatial cognition and memory deficits in ovariectomized (OVX) rats (Khaleghi et al., 2021). In addition, exogenous estrogen supplementation can improve anxiety–depression-like behavioral manifestations in OVX rats (Xu et al., 2016; Estrada et al., 2018; El-Khatib et al., 2020) and those with post-stroke depression (Jiang et al., 2021). The antidepressant effect of estrogen is mainly exerted by estrogen receptor (Walf et al., 2009; Furuta et al., 2013; Carrier et al., 2015). Estrogen act directly or indirectly through an estrogen-dependent mechanism. estrogen synthesis, metabolism and levels are altered during menopause, and estrogen acts as a potent ‘antidepressant’ by stabilizing these changes.

Some studies have reported that Sedentary lifestyle may be associated with increased levels of total cholesterol and high-density lipoprotein (HDL) of cardiovascular diseases, Increased risk of insulin and homeostatic model assessment of insulin resistance (HOMA-IR) in diabetes and increased levels of CRP and IL-6 in premature death (Biswas et al., 2015; Wirth et al., 2017). Considering that these are the leading causes of premature mortality among patients with depression, understanding and preventing excessive sedentary behavior is critical. A meta-analysis (Zhai et al., 2015) suggested that a sedentary lifestyle is associated with an increased risk of incident depression, and physical activity (PA) exerts the most protective effects against depression. Evidence suggests that 4 weeks of aerobic exercise significantly reduces the immobility time in the forced swim test (FST), increases sucrose preference and significantly reduces the immobility time and total distance travelled in the open field test (OFT) in rats with chronic unpredictable mild stress-induced post-stroke depression (Luo et al., 2019). Furthermore, 8 weeks of running training can significantly reduce the immobility time and increase the swimming and climbing time in FST in OVX mice or rats with chronic stress (Han et al., 2015; Kang et al., 2020; Tian et al., 2020) and increase the proportion of sucrose consumed (Tian et al., 2020). Additionally, 14 days of aerobic training can decrease the immobility time and increase the normal swimming time in FST in chronic unpredictable mild stress (CUMS)-induced rats (Kang et al., 2020). Notably, obesity is an established risk factor for the onset of depression (Luppino et al., 2010). Therefore, exercise may exert protective effects against depression by mitigating weight gain and the onset of obesity.

Bilateral ovariectomy is widely used to simulate the postmenopausal state of women (Diaz, 2012). Chronic restraint stress leads to persistent psychological stress (Seo et al., 2017; Son et al., 2019). However, studies on depression induced by both chronic restraint stress and ovarian hormone deficiency are lacking, and whether exercise exerts antidepressant effects *via* the estrogen receptor (ER) pathway remains unclear. Therefore, we hypothesized that ER signaling is a mechanism underlying the antidepressant effects of aerobic exercise. In this study, we found that female rats did not develop depressive symptoms shortly after ovariectomy; however, the susceptibility to depression increased in OVX rats with a gradual decrease in the levels of ovarian hormones. Restraint stress may accelerate the development of depressive disorders in OVX rats, and fluctuations in circulating estrogen levels after ovariectomy may lead to chronic restraint stress-induced depression. The objectives of the present study are as follows: (A) to observe and compare changes in the anxiety–depression-like and cognitive behaviors of female rats after short-term or long-term ovarian hormone depletion and long-term ovarian hormone deficiency combined with restraint stress intervention; (B) to observe and compare beneficial effects of estrogen and exercise training on anxiety–depression-like and cognitive behaviors in OVX rats with chronic stress; (C) to investigate whether ER antagonists can completely or partially counteract the positive effects of aerobic exercise.

## Materials and methods

### Animal management

Female Sprague–Dawley rats (age, 8 weeks) were purchased from the Guangdong Provincial Medical Laboratory Animal Center. The rats were housed (*n* = 4 rats/cage) under controlled temperature and light/dark environmental conditions (temperature, 23 ± 1°C; 12-h light/dark cycle) with free access to food and water. All animal experiments were approved by the Animal Protection and Use Committee of South China Normal University and conformed to the current animal welfare guidelines (Approval No. SCNU-SPT-2022-017). Significant efforts were made to minimize the number of animals used and their suffering or distress.

### Experimental procedure

After 4 weeks of acclimatization, at the end of 12 weeks of age, female rats (body weight, 284 ± 18 g) underwent bilateral oophorectomy or Sham surgery under pentobarbital (50 mg/kg) anesthesia.

**Figure 1:**
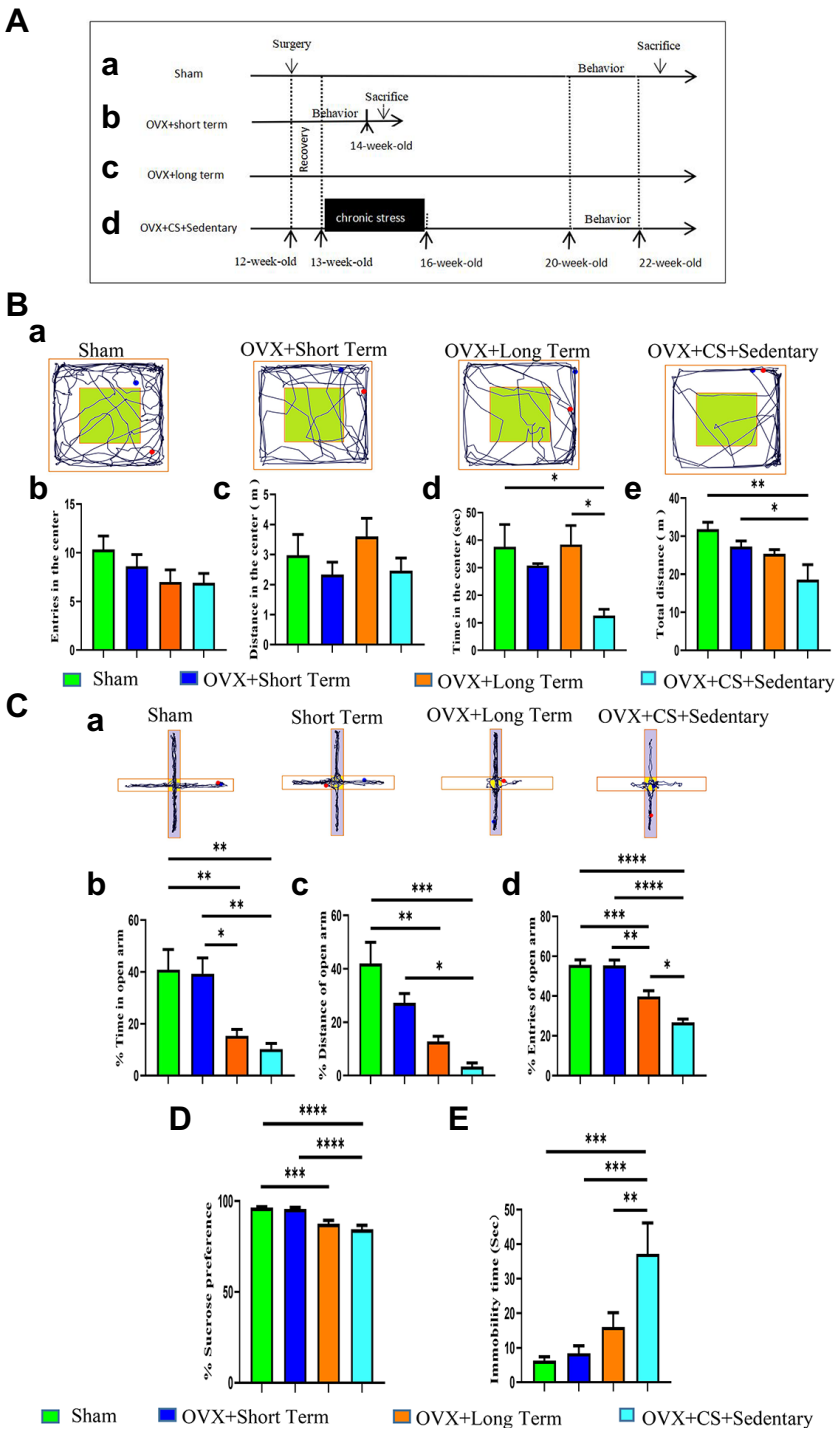
Experimental design **(A)** and ovariectomy accelerates the development of chronic stress-induced anxiety-depression-like behaviors **(B**,**C)**. **(A)** After 4 weeks of adaptive rearing, Sham surgery and bilateral ovariectomy (OVX) were performed on all 13-week-old female SD rats. Chronic restraint stress (CS, 7.5–8 h/day, 0:00-7:30/8:00, for 21 days). Pharmacological or exercise interventions were performed after completion of the stress protocol. behavioral tests (OFT, EPM, SPT, 3-chamber, BM, FST) of rats in Sham group, OVX+Long term group and OVX+CS+Sedentary group were performed during 21–22 weeks of age. Behavioral tests of rats in the OVX+Short term group were performed at 14 weeks of age. **(B)** Representative movement trajectory plots of rats in each group in the open field test **(a)**. The number of times rats entered the central area **(b)**, the time spent moving in the central area **(c)**, the distance moved in the central area **(d)**, and the total distance moved in the open field area **(e)** in the open field test. **(C)** Representative activity trajectories of rats in the elevated cross maze for each group **(a)**. The percentage of time rats spent moving on the open arm (white area) **(b)**, the percentage of distance moved on the open arm **(c)**, and the percentage of times they entered the open arm area **(d)**. **(D)** Percentage of sugar water preference in the sugar water preference test, sugar water intake as a percentage of total fluid intake. **(E)** Duration of immobility in the forced swim test. The cumulative time of immobility was analyzed. All values are expressed as mean ± SEM. *P < 0.05, **P < 0.01, ***P < 0.001, ****P < 0.0001.

The first part of the experiment (as shown in Figure 1A) was divided into four groups: sham (Sham, *n* = 10) group, estrogen short-term consumption group (OVX + Short term, *n* = 10) group, estrogen long-term consumption group (OVX + Long term, *n* = 10) group, and ovariectomy combined with chronic restraint stress group (OVX + CS + Sedentary, *n* = 12). Among them, rats in the OVX + CS + Sedentary group were subjected to chronic restraint stress 1 week after surgical recovery (weeks 14–16). Rats in the OVX + Short term group were scheduled for behavioral testing in the middle to week 14 of week 13. The other three groups of rats were scheduled to undergo behavioral testing at weeks 21–22.

**Figure 3:**
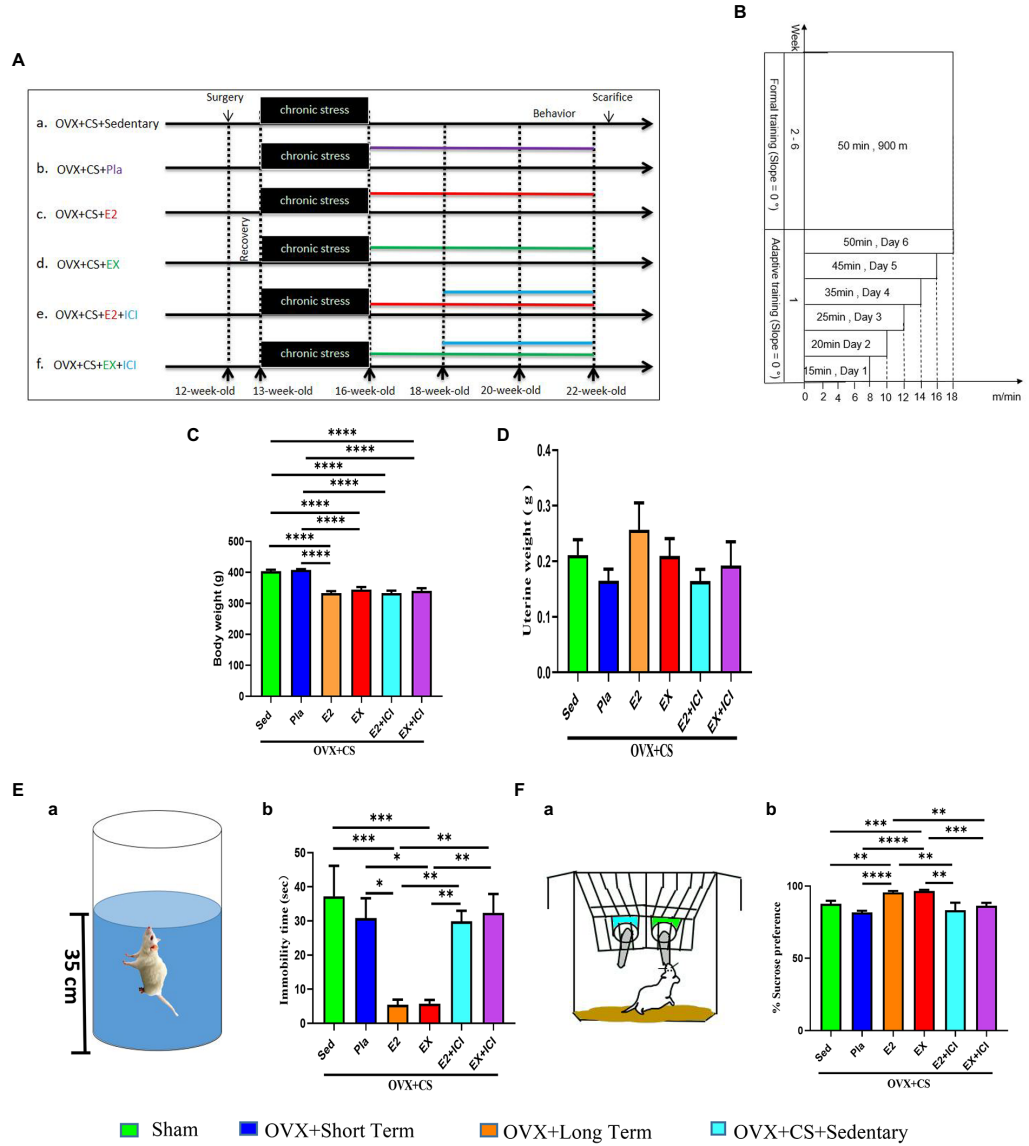
Estrogen and aerobic exercise effectively improved the depressive-like phenotype in depressed rats, but estrogen receptor antagonists reversed the antidepressant effect of aerobic exercise. **(A)** Experimental design. After the end of depression modeling, estrogen was administered (0.5 mM, 40 ug/Kg BW/day, ip) for a total of 6 weeks and antagonist was administered (10 μM, 10 mg/Kg BW/day, ip) for a total of 4 weeks. **(B)** Exercise program. The first week is the acclimatization training, the first day of training is 8 m/min, 0°, 15 min, after that the running speed is increased by 2 m/min per day, the exercise time is 5–10 min per day progressive increments, exercise acclimatization on the sixth day to 18 m/min, 0°, 50 min. Rest day on Sunday. The second week to the sixth week is the official training period, running platform program finally set at 18 m/min, 0°, 50 min. Body weight **(C)** and uterine weight **(D)** of rats in each group. **(E)** Schematic diagram of the sugar-water preference test **(a)** and percentage of sugar-water preference for statistical analysis **(b)**. **(F)** Schematic diagram of the forced swimming test **(a)** and immobility time **(b)**. All data are expressed as mean ± SEM (*n* = 8–12). *P < 0.05, **P < 0.05, ***P < 0.001, ****P < 0.0001.

The second part (shown in Figure 3A) was divided into ovariectomy combined with chronic restraint stress (OVX + CS + Sedentary, *n* = 12) group, placebo (OVX + CS + Placebo, *n* = 11) group, estrogen therapy (OVX + CS + E2, *n* = 11) group, exercise therapy (OVX + CS + EX, *n* = 11) group, estrogen plus estrogen receptor antagonist administration group (OVX + CS + E2 + ICI, *n* = 10), Exercise combined with estrogen receptor antagonist (OVX + CS + EX+ICI, *n* = 9) group. The rats in this part of the experiment were subjected to six behavioral tests sequentially at weeks 21–22.

### Surgical procedure

The first and second parts of the experiment were carried out sequentially, both at the end of 12 weeks of age and at the beginning of 13 weeks of age when the female rats were subjected to bilateral ovariectomy. The ovaries of rats were removed *via* bilateral ovariectomy (one finger next to the dorsal midline of the rat), and a longitudinal incision, approximately 1–1.5 cm long, was made around the midpoint of the ilium and rib cage. The skin and subcutaneous fascia were cut, the abdominal muscle was cut at the edge of the erector spinal muscle to enter the abdominal cavity and the fat was lifted. After the pink ovary was visible, the blood vessels and underlying fat were sutured, and the uterus was removed (Zhang et al., 2019). When closing the incision, caution should be observed to close the incision in layers, suturing the muscle layer first and then the skin, and iodophor disinfectant should be dabbed at the end of the suture. The incision will heal approximately 1 week after surgery.

### Chronic restraint stress

The chronic restraint stress program was started only after the surgical operation was completed. Chronic restraint stress is widely used to induce depression-like behaviors (Feng et al., 2019; Seewoo et al., 2020; Peng et al., 2021). Because rats are nocturnal animals, a nocturnal chronic restraint stress protocol (00:00–7:30/8:00 h; 7.5–8 h/day for 21 days) was used to limit their nocturnal behavior. Specifically, the rats were confined to a plastic bottle of the same size as the rat, and normal ventilation and defecation were ensured (Peng et al., 2021). After the procedure was completed, the rats were returned to their normal cages for feeding and drinking.

### Aerobic exercise

Based on a study by Bedford et al. (1979), the aerobic exercise routine was optimized with a pre-laboratory exercise protocol (Figure 3B). The first week of the exercise routine included acclimatization, and the running speed on the first day of acclimatization was 8 m/min at 0° for 15 min, after which the running speed was increased by 2 m/min every day and the exercise time was increased by 5–10 min, reaching up to 18 m/min, 0°, for 50 min on the sixth day of acclimatization, with rest on Sunday. In the second week of formal exercise, the running speed was 18 m/min, 50 min/day, 6 days/week at 0° for 5 weeks, with an intensity of 50–60% of the maximum oxygen uptake. Warm-up exercises were performed for 5–10 min before beginning the main exercise routine, and adjustment exercises were performed for 5–10 min after the end of the exercise routine.

### Drug administration

A 17β-estradiol was dissolved in 20% β-cyclodextrin (β-cyclodextrin was mixed with double-distilled water to make 20% β-cyclodextrin) at a final concentration of 0.5 mM (Lee et al., 2005;Ahn et al., 2011; Nag and Mokha, 2014). And administered subcutaneously at the dose of 40 μg/kg Body Weight/day for 6 weeks. Fulvestrant, an ER antagonist, was dissolved in corn oil and 20% dimethyl sulfoxide at a final concentration of 10 μM (Ahn et al., 2011; Nag and Mokha, 2014) and administered subcutaneously at the dose of 10 mg/kg of body weight/day for 4 weeks.

### Behavioral tests

#### Open field test

Open field test is a method for evaluating autonomous behavior, exploratory behavior and level of nervousness and anxiety in experimental animals in a novel environment (Wu et al., 2018). In a quiet environment with appropriate light intensity, rats were placed in a black, four-sided, flat box (100 cm × 100 cm × 40 cm), which was divided into 16 squares (4 cm × 4 cm). The video recorder and timer were stopped after 5 min of observation, and both rats and the inner walls and bottom of the box were cleaned with 75% alcohol to avoid the effects of their residues (e.g., urine, stool, and odor) on the results of the next test. The number of times the rats entered the central area, the time they stayed in the central area and the distance they moved in the central area within 5 min were recorded and analyzed using the ANY-maze software (Stoelting; Wood Dale, IL, United States).

#### Elevated plus maze

The elevated plus maze (EPM) test is a method for examining the anxiety-like state of animals based on their exploratory nature in a novel environment and their fear of high open arms to form conflicting behavior. The protocol of the EPM test used in this study was adapted from a previous experimental method (Pellow et al., 1985). The elevated cross maze has a pair of open arms (50 cm * 10 cm) and a pair of closed arms (50 cm * 10 cm). The rats are placed in the maze from the central grid surface (10 cm * 10 cm) toward the closed arms at the beginning of the experiment. The ANY-maze software (Stoelting; Wood Dale, IL, United States) was used to evaluate and record the number of entries into the open arm, the dwell time in the open arm, and the number of entries and dwell time in the closed arm in 5 min. The proportion of time spent in the open arm and the number of times the rats entered the open arm were calculated. At the end of the experiment, the rats were removed, their arms were cleaned and alcohol was sprayed to remove the odor.

#### Sucrose preference test

The sucrose preference test (SPT; Peng et al., 2021) is a reward-based test used as an indicator of a lack of pleasure. Briefly, the rats were housed in acrylic cages, and each cage was provided with two drinking bottles of identical shape and volume. One bottle contained drinking water, whereas the other bottle contained 1% sucrose solution. During the first 3 days of the test, both bottles contained equal volumes of drinking water for 24 h on the first day and 24 h on the second day. On the third day, the rat was not allowed access to food and water for 24 h. During the first 12 h of the fourth day, fasted and given equal volumes of both drinking water and 1% sucrose solution. The two bottles were removed after 12 h and weighed to calculate the consumption of sucrose water and drinking water. In addition, the positions of the two bottles were switched daily to avoid any confounders generated owing to the lateral bias. Sucrose preference was calculated as the percentage of sucrose intake relative to the total volume of fluid ingested.

#### Three-chamber social test

The three-chamber social test is based on the natural tendency of rats to live in groups and explore new objects (Silverman et al., 2013). Briefly, a three-chambered box (each chamber measuring 40 cm × 45 cm) was created by separating the chambers with clear Plexiglas, and a channel was created between chambers for the passage of rats. A metal cage was placed in the middle of the left and right chambers. The size of the cage should be large enough to accommodate an adult rat. The whole test process was divided into two stages, in the first stage (assessing the social novelty of test rats), an unfamiliar homozygous rat, Stranger 1, was placed in either of the side chambers (left or right chamber), whereas an empty cage was kept in the other side chamber. The ANY-maze software was used to evaluate and record the number of entries into each chamber, activity time and the distance travelled by the test rat in 10 min. In the second stage (assessing the social preferences of test rats), another unfamiliar rat of the same species, Stranger 2, was placed inside the empty metal cage, and the number of entries into each chamber, activity time and the distance travelled by the test rat in 10 min were evaluated and recorded using the ANY-maze software. After each round of the experiment, the inside of the cage and the floor were cleaned with 75% alcohol.

#### Barnes maze test

The Barnes maze (BM) test (Wu et al., 2020) is used to test the spatial memory of rodents by taking advantage of their light and dark avoidance and exploratory nature. The test procedure included a 3-day spatial exploration training period and a 1-day target localization test period. At the beginning of the training period, the rats were placed in a black opaque plastic drum in the center of the maze for 30 s. Thereafter, the drum was removed, and the rats were exposed to bright light, and latency to find the escape box and the number of errors in finding the box within 3 min were evaluated and recorded using the ANY-maze software. If a rat found and entered the escape box within 3 min, it was allowed to stay in the box for 30 s. If the rat failed to find the escape box, it was artificially guided to find and enter the escape box and was allowed to stay in the box for 30 s. During the fourth day of memory mapping, the escape box was removed from its original location, and latency to find the original escape box location and the exploration time in the target quadrant (where the escape box was located) during the 3-min session was evaluated and recorded using the ANY-maze software. After each trial, the maze and escape box were cleaned with 75% alcohol to eliminate residual odor, which can serve as a guide for the next animal.

#### Forced swim test

Forced swim test is also known as the behavioral despair experiment (Cryan et al., 2005; Peng et al., 2021). Forced swimming barrels were made of columnar, highly transparent acrylic Plexiglas, with a diameter of 30 cm and a height of 60 cm. The barrels were filled with water at a temperature of 27°C and a level of 35 cm. The cumulative immobility time of the rats after 4 min in the water during the 6 min test was recorded.

### Statistical analysis

The GraphPad Prism 8.3 software (GraphPad Software, Inc., San Diego, CA, United States) was used to statistically analyze and visualize the results of the behavioral tests. One-way analysis of variance (ANOVA) and Tukey’s *post hoc* test were used to compare dependent variables among multiple groups. Data are expressed as mean ± SEM for all tests, with *p*-values of <0.05 indicating significant difference.

This paper analyzed the quantitative difference (QD) (Liu et al., 2017; Guo and Liu, 2022) of each parameter instead of analyzing the qualitative difference in terms of the p-value. The QD was the absolute value of the golden logarithm of the ratio of two values of a parameter. There are three thresholds of the QD, (α, β, γ), i.e., (0.268, 0.805, 1.221), at neuropsychological or cellular and molecular levels, with QD<α indicating no difference (or called its as be biologically conserved) and QD<β indicating not significantly different(or called its as the plateau period of the curve in the Arndt-Schulz law) and β≤QD<γ indicating significant difference and QD≥γ indicating extraordinary significant difference. This paper then integrated all the parameters of a complex system in terms of their geometric mean which was called golden center (GC) and the GC of the Yin and Yang (GCYY) instead of studying the parameters one by one (Guo and Liu, 2022). If the GC was conserved, all the parameters can be classified into Yin and Yang parameters, which decrease and increase from an ill state to a healthy state, respectively, so that the Yin and Yang parameters were balanced, and the geometric mean of all the Yang parameters and all the inverse values of the Yin parameters is GCYY. GCYY based on the conserved GC can quantitatively characterize health states in terms of the QD threshold β or γ, with GCYY QD≥β indicating a quantitative criterion between a healthy state and its state of suboptimal health and GCYY QD≥γ indicating a quantitative criterion between a healthy state and its state suffering from a disease.

## Results

### Prolonged decrease in ovarian hormone levels accelerated the progression of depressive behavior in rats with chronic restraint stress

Open field test was used to evaluate the level of anxiety in rats. No significant differences were observed in the number of entries into the central area (*p* > 0.05; Figure 1Bb) and distance travelled (*p >* 0.05; Figure 1Bc) between the two groups. However, compared with OVX rats and Sham-operated rats, OVX rats with chronic restraint stress spent significantly less time in the central area (*p <* 0.05; Figure 1Bd). In addition, the total distance travelled in the open field area by OVX rats with chronic stress was significantly shorter than that travelled by Sham-operated rats and those with short-term estrogen depletion (STED; *p* < 0.05; Figure 1Be). These results indicated that anxiety-like behavioral were more pronounced in OVX rats with chronic restraint stress than in rats in the other two groups.

The anxiety-like behavioral performance of rats was further examined *via* the EPM test. The performance of rats with STED and Sham-operated rats was similar, with no significant differences (*p >* 0.05; Figures 1Cb–d). This result suggests that STED does not cause anxiety in rats. Furthermore, the time spent in the open arm was significantly less and the number of entries into the open arm was significantly lower among rats with long-term estrogen depletion (LTED) than among rats with STED (*p* < 0.05; Figures 1Cb,d), indicating that LTED leads to anxiety-like behavior in rats. In addition, the number of entries into the open arm was significantly lower among OVX rats with chronic stress than among rats with LTED (*p <* 0.05; Figure 1Cd), indicating that long-term ovarian dysfunction accelerates chronic restraint stress-induced anxiety-like behavior.

SPT was used to determine the lack of pleasure. Sucrose preference was significantly lower in both rats with LTED and OVX rats with chronic restraint stress than in Sham-operated rats (Figure 1D; *p* < 0.001). FST was used to test the depression-like behavior of rats. The immobility time was significantly longer in OVX rats with chronic restraint stress than in rats with LTED (Figure 1E*; p* < 0.01). These results indicate that long-term ovarian dysfunction accelerates the development of depression induced by chronic restraint stress.

### Low levels of circulating estrogen accelerated the development of social dysfunction induced by chronic restraint stress

**Figure 2:**
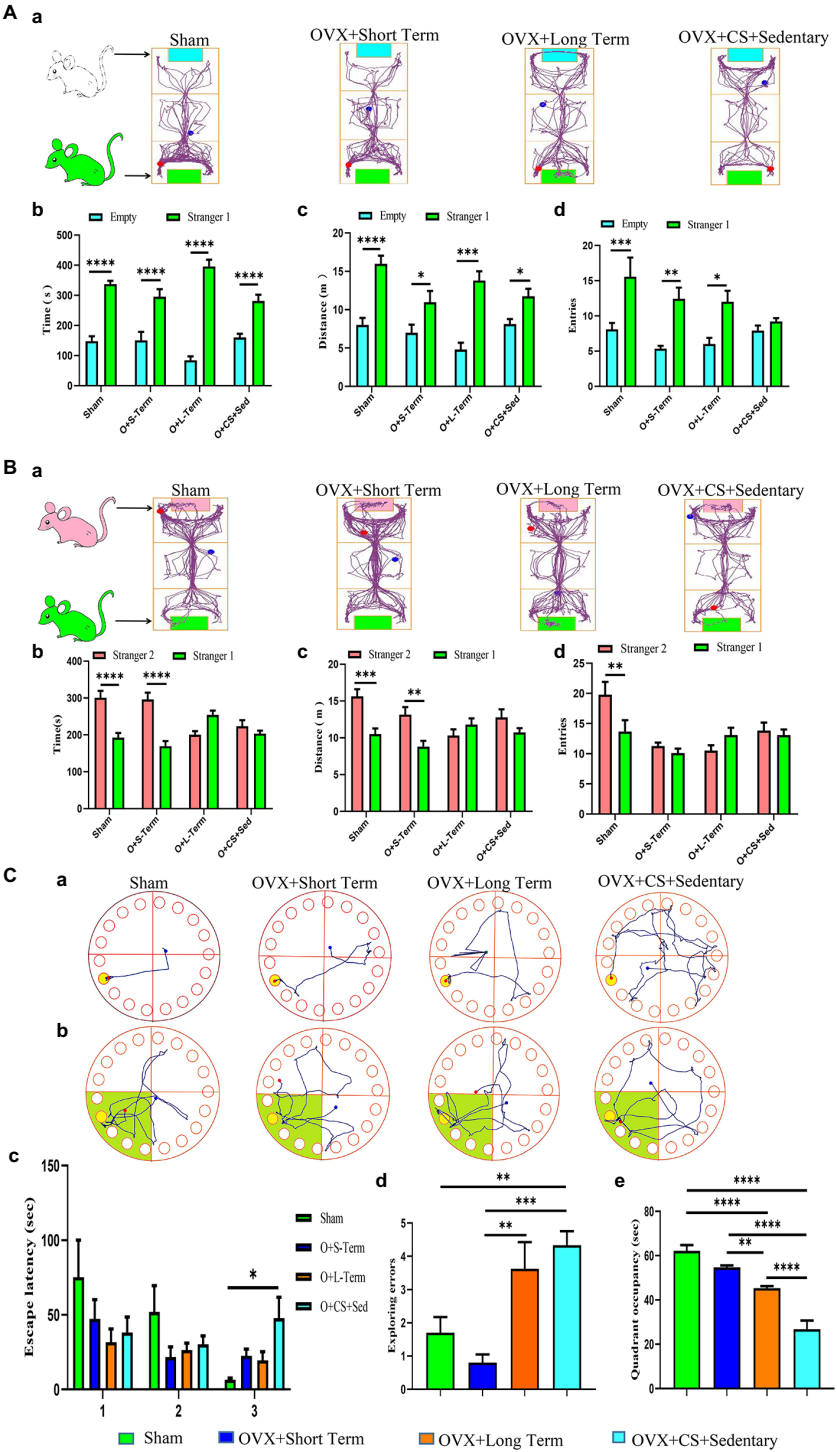
Ovariectomy accelerates the development of chronic stress-induced deficits in social functioning and spatial learning memory. **(A)** Representative locomotor trajectory plots of each group of rats in the three-chamber socialization test session I **(a)**. Duration of activity **(b)**, distance of activity **(c)**, and number of entries **(e)** of test rats in empty area or stranger 1 area. **(B)** Representative activity trajectories of each group of rats in the three-chamber social test Session II **(a)**. Activity time **(b)**, activity distance **(c)**, and number of entries **(e)** of the test rats in the stranger 2 area or the stranger 1 area. **(C)** Representative movement trajectories of each group of rats during the training period **(a)** and exploration period **(b)** in the Barnes maze task. Analyses were performed to record latency time during the training period **(c)**, the number of errors in exploring the escape box during the exploration period **(d)**, and dwell time in the target quadrant **(e)**. All values are expressed as mean ± SEM. (*n* = 8–12), *P < 0.05, **P < 0.01, ***P < 0.001, ****P < 0.0001.

Social behavior disorders are the hallmark of many psychiatric disorders, including depression. No significant difference was observed between the number of entries into the empty chamber and the number of entries into the chamber with Stranger 1 among OVX rats with chronic stress (Figure 2Ad; *p* > 0.05). In addition, no significant differences were observed in the dwell time, distance travelled and the number of entries into the two chambers with Stranger 1 and Stranger 2 between rats with LTED and OVX rats with chronic stress (Figures 2Bb–d; *p* < 0.05). These results suggest that ovarian hormone depletion leads to social dysfunction in female rats and exacerbates social behavior deficits induced by chronic restraint stress.

### Low levels of circulating estrogen accelerated the development of social dysfunction induced by chronic restraint stress

Social behavior disorders are the hallmark of many psychiatric disorders, including depression. No significant difference was observed between the number of entries into the empty chamber and the number of entries into the chamber with Stranger 1 among OVX rats with chronic stress (Figure 2Ad; *p* > 0.05). In addition, no significant differences were observed in the dwell time, distance travelled and the number of entries into the two chambers with Stranger 1 and Stranger 2 between rats with LTED and OVX rats with chronic stress (Figures 2Bb–d; *p <* 0.05). These results suggest that ovarian hormone depletion leads to social dysfunction in female rats and exacerbates social behavior deficits induced by chronic restraint stress.

### Long-term estrogen depletion exacerbated chronic restraint stress-induced learning and memory deficits

The BM test was used to examine the spatial memory capacity of the rodent hippocampus. During training, latency to find the escape box was significantly higher among OVX rats with chronic restraint stress than among Sham-operated rats (Figure 2Cc; *p* < 0.05); however, it was not significantly different between rats with LTED and Sham-operated rats (Figure 2Cc; *p > 0*.*05*). During the exploration phase, the number of errors in finding the escape box was significantly higher among rats with LTED than among those with STED (Figure 2Cd, *p* < 0.01), suggesting that the stability of ovarian hormone levels is important for the learning ability of female rats. The number of errors in finding the escape box was significantly higher among OVX rats with chronic restraint stress than among Sham-operated rats and those with STED (Figure 2Cd, *p* < 0.01). In addition, the dwell time in the target quadrant was significantly less among OVX rats with chronic restraint stress than among rats with LTED (Figure 2Ce, *p <* 0.0001). These results suggest that continuous external stressful stimulation impairs the spatial memory capacity of the hippocampus in OVX rats and exacerbates cognitive-behavioral deficits.

### Both aerobic exercise and estrogen effectively improved anxiety–depression-like behavior in depressed rats, whereas the estrogen receptor antagonist reversed the antidepressant effects of aerobic exercise

Both estrogen therapy and exercise significantly prevented the onset of obesity among OVX rats with chronic restraint stress (Figure 3C*; p* < 0.0001), suggesting that maintaining normal levels of estrogen and aerobic exercise are effective methods for controlling weight. However, they did not affect uterine weight in OVX rats (Figure 3D*; p* > 0.05).

Compared with rats in the placebo group and OVX rats with chronic stress, rats subjected to estrogen therapy and exercise had significantly increased sucrose preference (Figure 3Fb; *p* < 0.01) and significantly shorter immobility time (Figure 3Eb; *p* < 0.05). These results suggest that estrogen and exercise alleviate depression-like behavioral disorders. However, these beneficial changes were reversed after treatment with the ER antagonist (Figure 3E-F; *p <* 0.01). These results indicate that the antidepressant effects of both estrogen and exercise are mediated by ERs.

**Figure 4:**
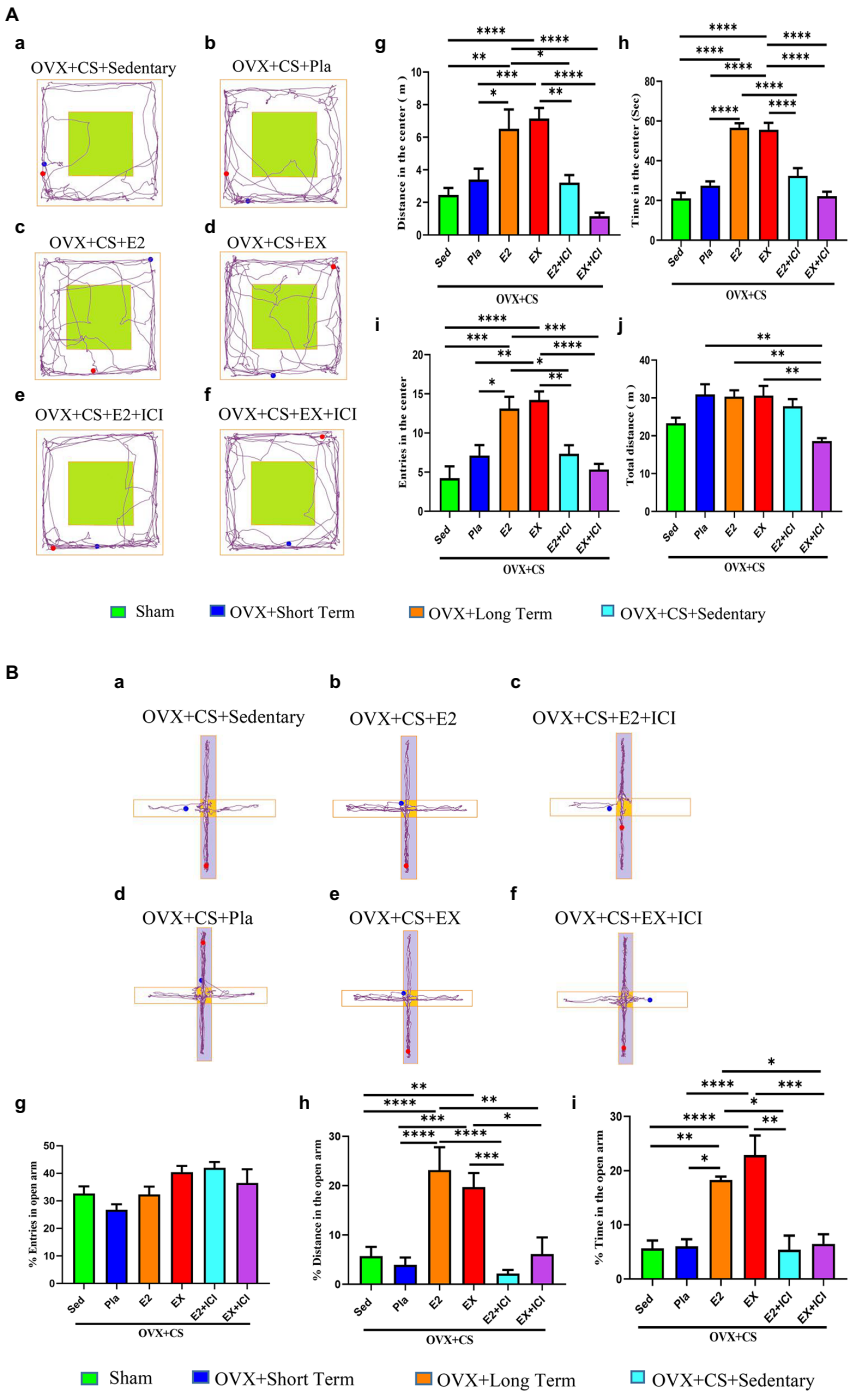
Estrogen and aerobic exercise improved the anxiety-like phenotype in depressed rats, but estrogen receptor antagonists blocked the anxiolytic effect of aerobic exercise. **(A)** Representative plots of activity trajectories of each group of rats in the open field test **(a–f)**. The activity distance **(g)**, activity time (h), number of entries **(i)** and total distance of activity in the open field **(j)** were analyzed for each group of rats in the central area of the open field. **(B)** Representative diagrams of the movement trajectories of rats in the elevated cross maze **(a–f)**. The percentage of times the rat entered the open-arm area **(g)**, the percentage of distance moved in the open-arm area **(h)** and the percentage of time spent moving **(i)** were analyzed. All data are expressed as mean ± SEM (*n* = 8–12). *P < 0.05, **P < 0.05, ***P < 0.001, ****P < 0.0001.

Estrogen treatment and exercise promoted changes in the anxiety-like behavior of OVX rats with chronic stress, which significantly increased the distance travelled (Figure 4Ag, *p* < 0.05), the time spent moving (Figure 4Ah, *p* < 0.0001) and the number of entries into the central area (Figure 4Ai *p* < 0.05) in OFT. Furthermore, in the EPM test, the distance travelled, and time spent in the open arm region were significantly higher among rats subjected to estrogen treatment and exercise than among rats in the placebo group and OVX rats with chronic restraint stress (Figures 4Bh,i, *p* < 0.05). These results suggest that exercise has effective antianxiety effects and may serve as an alternative to estrogen therapy.

### Aerobic exercise and estrogen treatment promoted the recovery of social function in depressed rats, whereas the estrogen receptor antagonist reversed the beneficial effects of aerobic exercise

**Figure 5:**
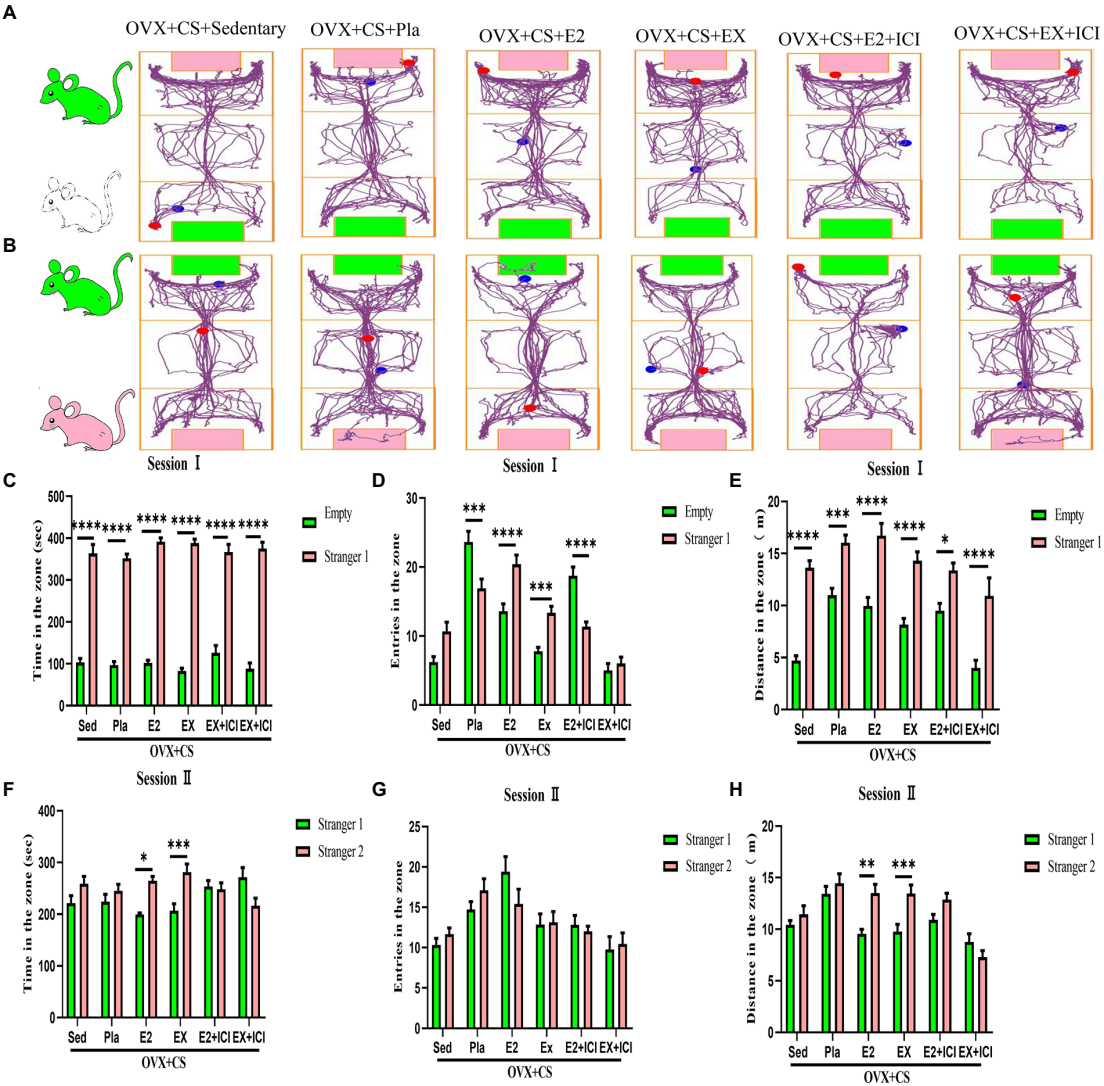
Both aerobic exercise and estrogen effectively promoted the recovery of social dysfunction in depressed rats, and the therapeutic effect of aerobic exercise was blocked by the administration of estrogen receptor antagonists. Representative plots of activity trajectories of each group of rats in the three-box social test session I **(A)** and session II **(B)**. The activity time **(C)**, number of entries **(D)**, and activity distance **(E)** in the Empty area and Stanger 1 area of the test rats were analyzed for intra- and inter-group comparisons. Intra- and inter-group differences in activity time **(F)**, number of entries **(G)**, and number of entries **(H)** were analyzed for the test rats in the stranger 1 area and the stranger 2 area. All data are expressed as mean ± SEM (*n* = 8–12). *P < 0.05, **P < 0.01, ***P < 0.001, ****P < 0.0001.

On assessing the social novelty of rats, as shown in Fig 5A, the rats in the OVX+CS +EX+ICI group spent significantly less time (Figure 5C, *p* < 0.0001) and moved significantly less distance (Figure 5E, *p* < 0.0001) in the area where the empty cage was located than in the area where Stranger 1 was located, but no significant difference was observed in the number of times the rats in the OVX+CS+EX+ICI group entered these two areas (Figure 5D, *p* > 0.05). Furthermore, on assessing social preference, as shown in Figure 5B, the dwell time and distance travelled were significantly different between depressed rats interacting with Stranger 1 and those interacting with Stranger 2 after estrogen therapy and exercise (Figures 5F–H, *p* < 0.05); however, this difference was disrupted after treatment with the ER antagonist (Figures 5F–H, *p* > 0.05). These results indicate that ER antagonists can prevent aerobic exercise-induced recovery of social dysfunction among depressed rats, suggesting that ERs mediate the antidepressant effects of aerobic exercise.

### Aerobic exercise and estrogen treatment significantly improved cognitive behavioral deficits in depressed rats; however, the estrogen receptor antagonist reversed the beneficial effects of aerobic exercise

**Figure 6:**
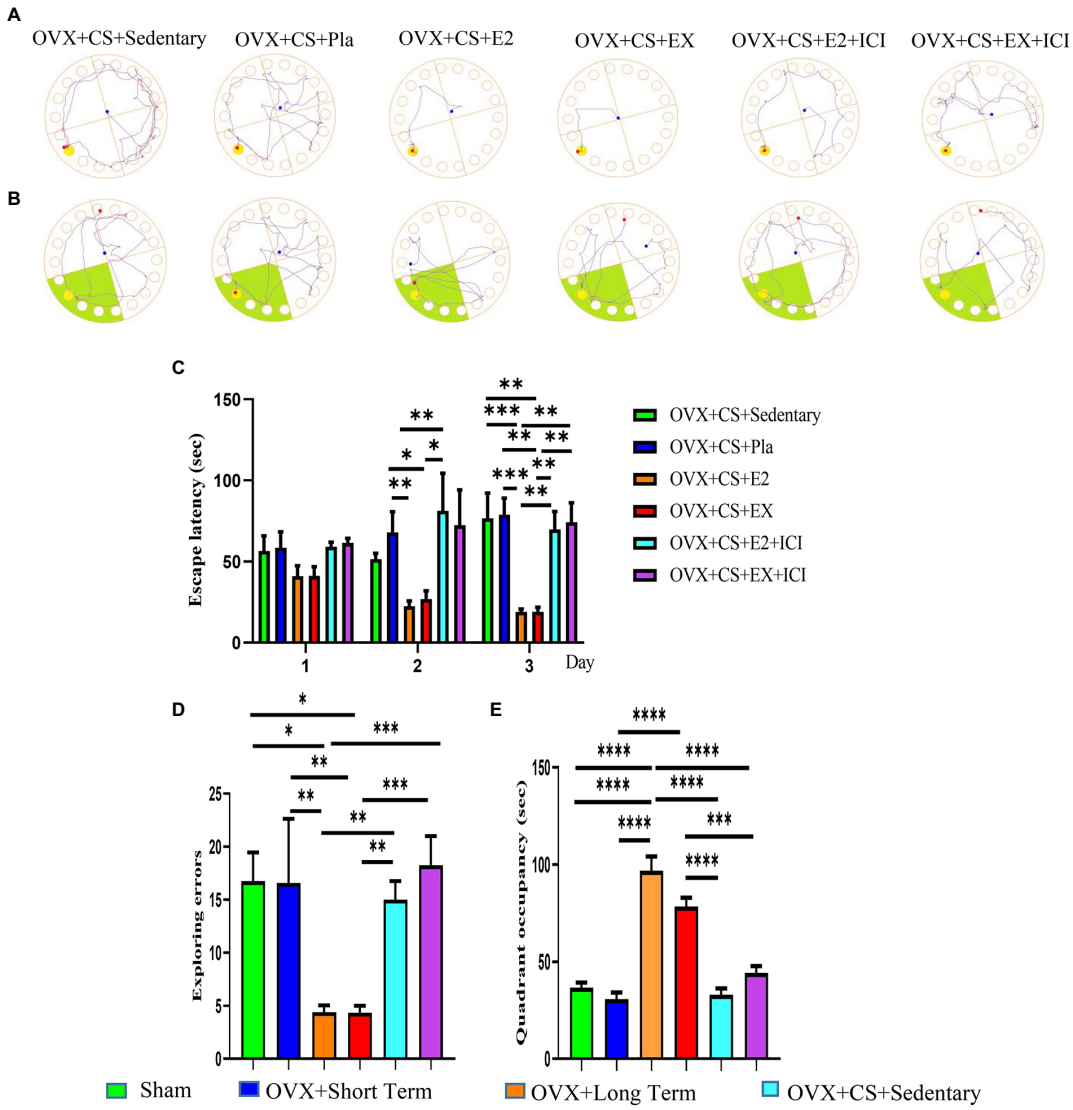
Both aerobic exercise and estrogen effectively regained learning memory function in depressed rats, while the beneficial treatment effects of aerobic exercise were inhibited by the administration of estrogen receptor antagonists. Representative graphs of the activity trajectories of each group of rats in the Barnes maze during the training period **(A)** and the exploration period **(B)**. Comparative analysis of latency time **(C)** within and between groups during the training period (days 1 to 3) and the number of errors in exploring the escape box **(D)**, and dwell time in the target quadrant **(E)** between groups 5during the exploration period. All data are expressed as mean ± SEM (*n* = 8–12). *P < 0.05, **P < 0.05, ***P < 0.001, ****P < 0.0001.

As demonstrated by representative traces in Figure 6A. Escape latency on the third day of the BM test was significantly lower among depressed rats subjected to estrogen therapy and exercise than among rats in the placebo group and those with depression (Figure 6C, *p* < 0.01). In addition Figure 6B, the number of errors on the fourth day of the test was significantly lower (Figure 6D, *p* < 0.01) and the dwell time in the target quadrant was significantly higher (Figure 6E, *p* < 0.0001) among depressed rats subjected to estrogen therapy and exercise than among rats in the placebo group and those with depression. These results suggest that both exercise and estrogen treatment effectively improve cognitive impairment in depressed rats, with comparable protective effects. Therefore, exercise may serve as an alternative to estrogen therapy as a healthy and non-toxic ‘antidepressant’. However, no difference in the above-mentioned parameters was observed between the OVX + CS + EX+ICI group and the OVX + CS + E2 + ICI group as well as between depressed rats and those in the placebo group (Figures 6C–E, *p > 0*.*05*). These results suggest that the protective effects of exercise on learning and memory are inhibited by ER antagonists, indicating that exercise exerts antidepressant effects by activating ERs.

### Quantitative and integrative analysis of behavioral data

The overall health (*via* quantitative characterization by GCYY value) of rats in each experimental group was observed and compared *via* GC (as shown in Table 1). The health scores of the Sham and STED groups were 10.919 and 10.147, which were high and similar, indicating that rats in these two groups were healthiest. The health scores of the exercise, estrogen treatment and LTED groups were 9.024, 8.819, and 7.577, respectively, which were similar to and second only to the scores of the two aforementioned healthiest experimental groups. These results indicate that exercise and estrogen can significantly improve the anxiety–depression-like behavior of depressed rats. Furthermore, rats in the LTED group did not develop depression. The health scores of the depression (4.742 or 4.008), placebo (4.509), and OVX + CS + EX+ICI or OVX + CS + E2 + ICI groups (4.472, 3.735) were lower. The health scores of the exercise, estrogen treatment and LTED groups were no significantly different from those of the Sham or STED group (QD < 0.805) but were significantly different than those of the depression, placebo and exercise or estrogen plus antagonist groups, which its significantly different reached extraordinary level (QD > 1.221).

**Table 1:** Quantitative difference statistics for the geometric mean of all behavioral data.

	Sham	OVX + Short Term	OVX + CS + EX	OVX + CS + E2	OVX + Long Term	OVX + CS + Sedentary	OVX + CS + Sedentary	OVX + CS + Pla	OVX + CS + E2 + ICI	OVX + CS + EX + ICI
Time in the center (sec)	52.528	51.337	54.238	56.161	49.011	14.466	15.734	28.792	35.059	21.689
Entries in the center	12.413	11.325	14.296	12.613	8.769	6.658	2.621	7.192	6.889	4.921
Distance in the center (m)	7.057	7.012	6.902	6.174	4.134	2.694	2.694	4.366	3.084	0.718
Total distance (m)	33.126	27.556	29.620	29.897	25.190	19.835	22.743	31.762	27.151	18.524
% Entries in the center	60.942	46.654	38.481	31.529	39.128	26.485	32.875	26.321	41.655	39.478
% Time in the open arm	32.734	28.844	18.993	18.220	16.314	13.767	4.064	4.594	3.716	2.499
% Distance in the open arm	26.033	24.805	16.933	20.070	13.733	3.084	1.911	1.249	1.355	1.434
% Sucrose preference	96.413	95.659	96.620	95.570	87.416	84.368	87.360	81.764	82.955	86.251
Immobility time (sec)	0.168	0.185	0.145	0.179	0.052	0.035	0.044	0.035	0.035	0.042
Escape latency (sec)-Day 1	51.788	40.413	38.524	38.629	22.898	23.195	46.712	57.488	58.628	61.331
Escape latency (sec)-Day 2	23.815	19.523	20.976	19.982	33.896	40.717	49.882	55.577	42.599	50.462
Escape latency (sec)-Day 3	0.077	0.080	0.049	0.055	0.066	0.023	0.022	0.014	0.019	0.016
Quadrant occupancy (sec)	82.699	75.666	77.418	93.937	45.284	32.373	35.937	33.111	31.180	43.365
Exploring errors	0.341	0.386	0.280	0.167	0.500	0.236	0.062	0.082	0.070	0.065
GCYY	10.919	10.147	9.024	8.819	7.577	4.742	4.008	4.509	4.472	3.735
QD (X vs. Sham)	0.000	0.152	0.396	0.444	0.759	1.733	2.082	1.838	1.855	2.229
QD (X vs. OVX + Short Term)	0.152	0.000	0.244	0.291	0.607	1.580	1.930	1.685	1.703	2.077

## Discussion

In this study, the short-and long-term effects of ovariectomy on the behavior of rats were examined. A previous study showed that the time spent in the central area in OFT, distance travelled and immobility time in FST were not different between the experimental and control groups when the duration of estrogen depletion was short (14–16 days; Khayum et al., 2020). Nakagawasai O et al. evaluated the immobility time in FST, time spent in the open-arm area in the EPM test and latency in the passive avoidance task in female mice 14 days after ovariectomy. No differences in these parameters were observed between OVX mice and mice in the Sham group (Nakagawasai et al., 2009). Another study showed that the immobility time in FST after 2, 4 and 8 weeks of ovariectomy was not different between OVX mice and control mice (Han et al., 2015). In this study, the results of six behavioral tests were similar in the STED and Sham groups. Depression does not develop shortly after ovariectomy because the levels of estrogen in the brain are relatively high 2 weeks after ovariectomy, which plays a protective role in stabilizing the mood (Chu et al., 1999). In addition, given the time required for ovarian hormone withdrawal, the increased susceptibility to depression caused by decreased ovarian hormone levels requires the accumulation of temporal effects.

According to the ‘critical period of estrogen neuroprotection’ hypothesis, the ability of E2 to exert neuroprotective effects in the CA1 region of the hippocampus is lost after 10 weeks of ovariectomy (Zhang et al., 2011; Scott et al., 2012). LTED severely decreases estrogen concentration (approximately 10% of the total premenopausal estrogen levels), which triggers anxiety-and depression-like behavioral disorders and cognitive impairment. Several studies have validated this causal relationship (Fedotova et al., 2017; Dornellas et al., 2018; Campos et al., 2020; Banin et al., 2021). In the present study, rats with LTED did not exhibit anxiety-like behavior in OFT within 10 weeks of ovariectomy. However, the dwell time and distance travelled were shorter and the number of entries into the open arm was lower in the EPM test among rats with LTED than among control rats, suggesting that LTED induces some degree of anxiety-like behavior. Depressed rats (OVX + CS + sedentary group) spent less time in the open arm and entered the open arm less frequently in the EPM test than rats with LTED. In addition, they exhibited sluggish behavior in the central region in OFT. Therefore, depressed rats develop more severe anxiety-like behavior than LTED rats after the same amount of time. Sucrose preference was significantly reduced in rats with LTED, and depressed rats compared with rats in the Sham group, suggesting that LTED and/or stress reduced the ability of rats to feel pleasure, that is, the rats are no longer sensitive to the sweet tooth regulated by reward mechanisms. In FST, the immobility time was not significantly different between the LTED and Sham groups but was longer in depressed rats than in rats with LTED, which is consistent with the results of previous studies (Han et al., 2015). These results indicate that LTED induces some degree of depression-like behavior in rats, whereas LTED combined with stress can induce severe depression-like behavior. Therefore, low levels of circulating ovarian hormones contribute to the susceptibility of rats to depression and exacerbate stress-induced anxiety–depression-like behavior in a hormone level-dependent manner (Takuma et al., 2012; Eid et al., 2020; Ge et al., 2020; Jing et al., 2020).

In session II of the three-chamber social test, the contact time, activity distance and number of entries into chambers were not different between LTED rats interacting with stranger 1 and those interacting with stranger 2, indicating that LTED induces social behavior deficits. Depressed rats showed significant deficits in both social novelty and preference, and more severe social behavior deficits were observed in depressed rats than in those with LTED. This finding is similar to that of another study in which test rats less frequently entered the chamber with stranger 1 and had travelled shorter distances compared with control rats (Zain et al., 2019). These results suggest that chronic restraint stress impairs the socialization capability in rats, and LTED increases the susceptibility of rats to socialization. These changes may be related to decreased serum estradiol levels and increased corticosterone levels (Khaleghi et al., 2021). In the BM test in this study, the number of errors was higher and dwell time in the target quadrant was shorter among rats in the LTED group than among rats in the STED group. In addition, escape latency and the number of errors in finding the target hole were significantly higher among depressed rats than among rats in the Sham and STED groups. Depressed rats spent significantly less time in the target quadrant compared with rats in the other three groups. These results suggest that LTED, instead of STED, causes memory deficits in rats, and chronic restraint stress combined with chronic estrogen deprivation exacerbates learning and memory deficits in rats (Kim et al., 1996, 2015).

Rats with LTED were more anxious than rats with STED, suggesting that the severity of or susceptibility to anxiety increases with a progressive decrease in ovarian hormone levels. Furthermore, for 10 weeks, the stressful external environment further exacerbated anxiety and depression in OVX rats. Therefore, rats in the OVX + CS + sedentary group may have developed anxiety and depression before the end of 10 weeks, and we might not have detected these behaviors at the right time.

Unlike in previous studies (Hao et al., 2010; Thomas et al., 2010), estrogen treatment in this study did not significantly affect the uterine weight of depressed rats, however, it was marginally increased. Similarly, exercise did not significantly affect uterine weight. This phenomenon may be attributed to the significant response induced by chronic restraint stress, which occurs through neural (physiological) adaptation by activating the HPA axis and promoting the release of pro-adrenocorticotropic hormone, which generates an appetitive suppression response leading to reduced food intake and weight loss (van der Kooij, 2020). In addition, Corticotropin releasing hormone stimulates the sympathetic nervous system and catecholamine release, increasing the thermogenesis and lipolysis of brown adipose tissue and inhibiting the proliferation of preadipocytes (Razzoli et al., 2015), leading to weight loss.

In this study, compared with rats in the depression and placebo groups, those treated with estrogen showed significant improvements in anxiety–depression-like behavior, social novelty preference and hippocampal spatial memory. This finding is consistent with that of previous studies on the antidepressant effects of estradiol (Takuma et al., 2007; Récamier-Carballo et al., 2012; Rashidy-Pour et al., 2019). Although estrogen replacement therapy can reduce anxiety and depression, it requires strict and individualized dosing regimens and schedules; otherwise, there is a risk of developing breast and endometrial cancers (Ravn et al., 1994; Ayres de Campos et al., 1997). Regular physical activity is a well-known non-pharmacological neuroprotective method (Carek et al., 2011) and has been widely certified for its beneficial modulation of cognitive function and emotional behavior of the brain (Archer et al., 2014). In this study, OVX rats with chronic stress-induced depression showed significant improvements in anxiety–depression-like behavior and social, learning and memory abilities after 6 weeks of aerobic training, which is consistent with the findings of previous studies (Lu et al., 2014; Kim and Leem, 2016; Xu et al., 2016; Leem et al., 2019). Notably, no significant overall difference was observed between the antidepressant effects of aerobic exercise and estrogen therapy.

Estrogen regulates mood and endocrine homeostasis mainly by binding to ERs in the brain. The ER antagonist ICI 182780, called fulvestrant, used in this study has a high binding affinity for ERs and blocks the nuclear localization of ERs by impairing receptor dimerization and energy-dependent nucleoplasm transport, which reduces intracellular ER levels and blocks ER-mediated gene transcription (Howell, 2006). ER antagonist intervention can decrease the expression of ERα and ERβ in neurons (Xu et al., 2020), block the biological effects of ER-mediated estradiol and inhibit the neuroprotective effects of estrogen. In this study, the ER antagonist fulvestrant inhibited the antidepressant effects of estrogen replacement therapy, which is consistent with the results of previous studies (Xu et al., 2016; Estrada et al., 2018; Eid et al., 2020). Moreover, fulvestrant also inhibited the antidepressant effects of aerobic exercise, which has not been reported in previous studies. This suggest that exercise may exert antidepressant effects through ERs.

Quantitative differences are examined by treating living phenomena as a system, that is, as a whole, which is consistent with the organismic concept in biology (von Bertalanffy, 1926; Drack et al., 2007). A system is an organic whole with a certain structure and function composed of interconnected and interacting elements (parts). The golden center (GC; Guo and Liu, 2022), also known as the geometric mean of all the parameters, is a quantitative and integrative method used in statistics to integrate all the parameters (in this case, behavioral parameters) of a complex system, while the GC of the Yin and Yang (GCYY) based on the conserved GC can be used to evaluate a healthy state of all parameters integrated together (Guo and Liu, 2022). In this study, all behavioral parameters were integrated into one dataset, and each experimental group was considered a subsystem for evaluating and comparing the strength of anxiety–depression-like behavior of rats in different experimental groups. Rats in the STED and Sham groups did not exhibit depressive behavior. Rats in the exercise, estrogen treatment and LTED groups had lower levels of vitality than the aforementioned two groups in terms of anxiety and depression, that is, these rats were in a sub-healthy state. Rats in the depression and OVX + CS + EX+ICI and OVX + CS + E2 + ICI groups had the lowest vitality owing to severe anxiety and depression, which further indicates the key finding of this study: ERs may mediate the antidepressant effects of aerobic exercise.

After ovariectomy, the mRNA expression of ERα and ERβ in the hippocampus of OVX rats decreased with a progressive decrease in estrogen levels (El-Khatib et al., 2020). Some studies have shown that improvement in depression-like behavior in OVX rats is partly attributed to the increased serum E2 levels after exercise (Lu et al., 2014), whereas several studies have reported no increase in plasma or serum estrogen levels in OVX rats after exercise (Choi et al., 2005; Rauf et al., 2015; He et al., 2021). Notably, a study showed that cerebellar estrogen levels were significantly higher after exercise (Rauf et al., 2015). Therefore, the antidepressant effects of aerobic exercise may be attributed to the continued activation of nuclear ERs *via* local brain-derived estrogen synthesis. In addition, the C-terminal end of the E3 ubiquitin ligase of heat shock homologous protein 70 (Hsc70)-interacting protein (CHIP) can bind to the unbound ERα and target it for ubiquitination and proteasomal degradation (Tateishi et al., 2004; Fan et al., 2005; Baumgartner et al., 2021). Long-term ovarian hormone deprivation after OVX enhances the interaction between rat hippocampal ERα and CHIP, thereby accelerating the ubiquitination of ERα for degradation (Zhang et al., 2011). Therefore, exercise may also exert antidepressant effects by blocking ER degradation.

IGF-I can cross the blood–brain barrier, is a pleiotropic neuroprotective signal (Fernandez and Torres-Alemán, 2012; Zhou et al., 2022) and has powerful mood-and cognitive-modulating effects (Aleman and Torres-Alemán, 2009; Munive et al., 2016). ERα appears to be a part of the IGF-I signaling machinery in the brain. IGF-I can activate ER in the absence of estradiol (Font de Mora and Brown, 2000; Martin et al., 2000; Klotz et al., 2002). Although detectable levels of neuroactive steroids are maintained in the nervous system of gonadectomized animals, and the levels of certain neuroactive steroids are increased (Caruso et al., 2010); however, ovariectomy eliminates the changes in IGF-I and its mRNA after exercise (Munive et al., 2016). The loss of the effects of exercise after menopause may be related to the loss of the interaction between IGF-IR and ERα in brain endothelial cells (Munive et al., 2019). Therefore, the response of IGF-I to exercise depends on intact ovarian function, that is, ovarian steroids are sensitive to exercise. In addition to 17β-estradiol and IGF-I, exercise may also regulate the transcriptional activity of ERα by activating intracellular kinase signaling pathways through other growth factors, which remain unknown.

## Conclusion

Long-term estrogen deprivation increases the risk of developing chronic restraint stress-induced depression. Aerobic exercise can significantly improve anxiety–depression-like behaviors, and ERs may mediate the antidepressant effects of aerobic exercise.

## Data availability statement

The raw data supporting the conclusions of this article will be made available by the authors, without undue reservation.

## Ethics statement

All animal experiments were approved by the Animal Protection and Use Committee of South China Normal University and conformed to the current animal welfare guidelines (Approval No. SCNU-SPT-2022-017). Significant efforts were made to minimize the number of animals used and their suffering or distress.

## Author contributions

RZ and TL designed the experiments. TL funded this experiment. RZ was responsible for conducting the experiments, processing and analyzing the data, and writing and revising the article. ZW provided help in revising and embellishing the article, and has introduced and written the statistical methods of GC, GCYY and QD in the quantitative and integrative analysis of data. BZ contributed directly to the revision and embellishment of the article and the submission of the manuscript. CW gave valuable comments on the refinement of the experimental design and the revision of the data plots. ZY, JH, and KC helped to carry out this experiment. All authors contributed to the article and approved the submitted version.

## Funding

This study was supported by the National Natural Science Foundation grants of China (11604104 and 61575065).

## Conflict of interest

The authors declare that the research was conducted in the absence of any commercial or financial relationships that could be construed as a potential conflict of interest.

## Publisher’s note

All claims expressed in this article are solely those of the authors and do not necessarily represent those of their affiliated organizations, or those of the publisher, the editors and the reviewers. Any product that may be evaluated in this article, or claim that may be made by its manufacturer, is not guaranteed or endorsed by the publisher.
